# Increased carotid intima–media thickness associated with high hs-CRP levels is a predictor of unstable coronary artery disease

**DOI:** 10.5830/CVJA-2013-061

**Published:** 2013-10

**Authors:** Sejran Ahmet Abdushi, Feim Durak Nazreku, Fadil Ukë Kryeziu

**Affiliations:** Prim Dr Daut Mustafa Regional Hospital, Prizren, Republic of Kosovo; Prim Dr Daut Mustafa Regional Hospital, Prizren, Republic of Kosovo; National Institute of Public Health, Prizren, Republic of Kosovo

**Keywords:** CIMT, hs-CRP, atherosclerosis, stable coronary disease, unstable coronary disease

## Abstract

**Abstract:**

Increased values of carotid intima–media thickness (CIMT) and high-sensitivity C-reactive protein (hs-CRP) are predictors of acute coronary events. We analysed the link between CIMT and hs-CRP in cases with coronary artery disease (CAD). From 1 January to 30 June 2012, we evaluated 43 patients with acute coronary syndrome (group A), 50 patients with stable coronary artery disease (group B) and 50 healthy volunteers (group C). All were analysed for CIMT and hs-CRP levels. CIMT values were higher in groups A and B (0.94 ± 0.21 mm, 0.89 ± 0.19 mm, respectively) and lower in group C (0.64 ± 0.09 mm), and this was statistically significant (*p* < 0.0001). However the values of hs-CRP were higher in group A (1.87 ± 0.36 mg/l) and lower in groups B and C (1.07 ± 0.28 mg/l, 0.97 ± 0.45 mg/l, respectively) and this was also statistically significant (*p* < 0.0001).

## Abstract

More than half of acute myocardial infarctions originate from blood vessels with stenosis of less than 50%.^1^ Moreover, cholesterol level is a poor predictor of cardiovascular risk. This was documented by data from the Framingham Heart study, where more than a third of patients with coronary artery disease (CAD) had values of total cholesterol lower than 5.1 mmol/l.^2^ A method is therefore needed to improve prediction of cardiovascular risk. During the 1990s it became clear that many other factors besides conventional risk factors, such as homeostatic and thrombotic mechanisms, markers of inflammation and genetic risk factors may have an influence on cardiovascular risk.^3^-^9^

For pathogenesis of coronary artery disease, the presence of atherosclerotic plaques is significant.^4^ The structure of the coronary artery wall is not static. With increase in its external diameter, development of atherosclerotic plaques will be possible without significant narrowing of the lumen of the artery.^10^ Several necropsy studies have reported very strong correlations between atherosclerosis in the carotid and coronary arteries.^11^,^12^

Increase in carotid artery intima–media thickness (CIMT) is considered a marker for early atherosclerosis.^13^ Risk prediction for coronary artery disease may be improved by additional information on the increase in CIMT, together with traditional risk factors.^14^

Recently, inflammation has emerged as an important factor in the process of atherosclerosis,^15^ therefore hs-CRP has been included as a new risk factor for CAD.^16^ In a recent study it was concluded that both hs-CRP and conventional lipid parameters can be used to predict the risk for CAD.^17^

Exercise stress testing provides useful information on the prognosis of patients with stable CAD and stable patients after acute coronary syndrome.^18^ Myers *et al.* found that subjects with stable CAD who achieved < 5 METs (metabolic equivalents) in exercise stress tests had four times higher mortality rates than subjects who achieved > 10 METs.^19^

The aim of this study was to analyse the association between changes in CIMT and hs-CRP values in cases with stable and unstable coronary artery disease.

## Methods

Between 1 January and 30 June 2012, a total of 143 subjects were included in this prospective study. All subjects were placed in three groups: group A (patients with acute coronary syndrome) included 43 patients with acute coronary artery syndrome, 25 (58.14%) with acute myocardial infarction and 18 (41.86%) with unstable angina pectoris. Group B included 50 patients with stable coronary artery disease, 37 (74%) of them with stable angina pectoris and 13 (26%) with a stable condition after myocardial infarction, and all achieved ≥ 5 METs in exercise stress testing. Group C (control group) included 50 healthy volunteers with negative exercise stress testing.

We excluded all subjects with acute infection, active chronic inflammatory diseases (inflammatory bowel disease, rheumatic diseases, upper and lower respiratory tract diseases, etc.), patients after a recent myocardial infarction (less than one month before the study onset), patients with recent trauma (surgery, burns) and those with malignancies. Adjustment was made for age and gender. All individuals were interviewed about risk factors and regularity of therapy.

Routine biochemical analyses were performed, with special emphasis on fasting glucose levels and lipid profiles [total cholesterol, low-density lipoprotein (LDL) cholesterol, highdensity lipoprotein (HDL) cholesterol and triglycerides]. In group A, blood samples for hs-CRP were obtained on admission, and at a time interval shorter than six hours from the onset of symptoms, and stored at –70°C. We also took samples for cardiac (troponin I, myoglobin, CK-MB) enzymes.

**Fig. 1.**
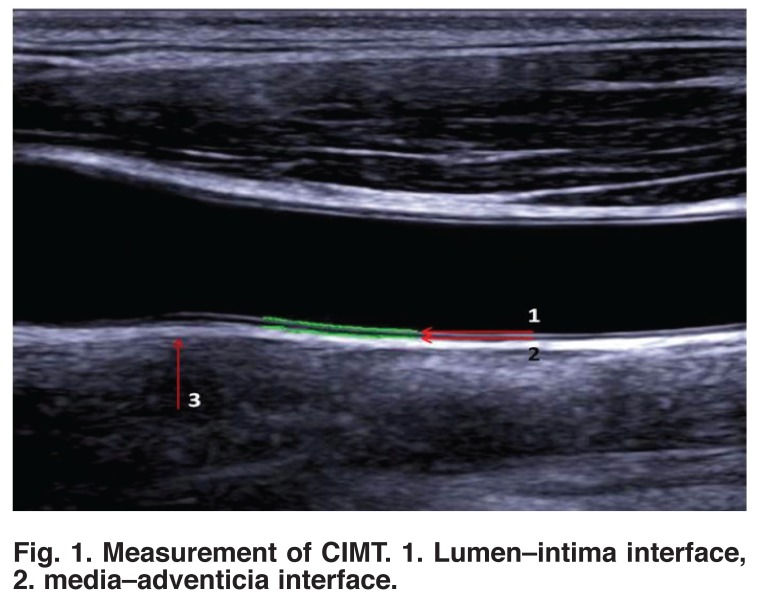
Measurement of CIMT. 1. Lumen–intima interface, 2. media–adventicia interface.

Carotid ultrasound was done by a single operator using an Aloka-Prosound SSD-4000SV system equipped with a 7.5-MHz linear array probe. Measurements were carried out at the far wall (far wall from ultrasound probe) on the right and left common carotid artery, as recommended by the American Society of Echocardiography 2008,^20^ and the mean value was used in the study (Fig. 1). Diagnosis of acute coronary syndrome was established on the recommendations of the European Society of Cardiology 2011^21^ and 2012.^22^

Bicycle exercise testing (Cardiax stress ECG, Germany) was performed to assess exercise functional capacity (expressed in METs) using the Bruce protocol.^23^ Exercise was continued until the heart rate reached 85% of the estimated maximum age-predicted target heart rate for each patient, or was symptom limited. We considered only patients who achieved ≥ 5 METs.

## Statistical analyses

All data are presented as the mean ± SD or frequency (%), unless otherwise stated. The baseline clinical characteristics of the groups were compared using one-way ANOVA for continuous variables and the chi-square test for non-continuous variables. Statistical significance was set at *p* < 0.05.

## Results

**Fig. 2.**
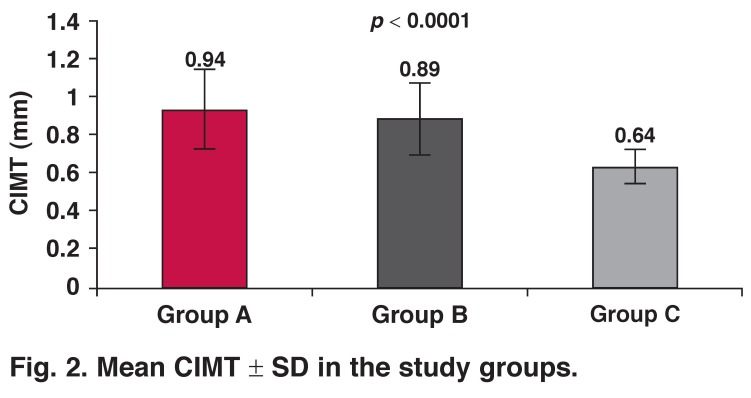
Mean CIMT ± SD in the study groups.

Baseline clinical characteristics of the study population are summarised in Table 1. CIMT values were higher in group A and B, and lower values were found in group C (0.94 ± 0.21 mm, 0.89 ± 0.19 mm, 0.64 ± 0.09 mm, respectively). Statistical analysis showed significant differences between groups A and C (*p* < 0.0001), and also between groups B and C (*p* < 0.0001), but no significant difference was found between groups A and B (*p* > 0.05) (Fig. 2).

**Table 1:** Baseline Clinical Characteristics Of The Study Populations

	*Group A*	*Group B*	*Group C*	p*-value*
Age (years)	59.3 ± 4.5	57.3 ± 9.7	56.1 ± 7.3	0.129 NS*
Male, *n* (%)	29 (67.44)	32 (64)	23 (46)	0.072 NS**
Hypertension, *n* (%)	18 (41.86)	17 (34)	9 (18)	0.038 S**
Diabetes, *n* (%)	22 (51.16)	22 (44)	11 (22)	0.022 S**
BMI (kg/m^2^)	29.37 ± 2.7	28.12 ± 2.3	24.6 ± 3.1	0.0001 S*
Fasting glucose (mmol/l)	7.3 ± 2.15	6.94 ± 1.81	5.733 ± 2.29	0.001 S*
Total cholesterol (mmol/l)	6.45 ± 2.31	6.13 ± 2.10	5.13 ± 1.48	0.004 S*
LDL-C (mmol/l)	4.53 ± 1.27	4.1 ± 1.01	2.97 ± 1.11	0.0001 S*
HDL-C (mmol/l)	0.95 ± 0.32	1.05 ± 0.29	1.41 ± 0.34	0.0001 S*
Triglycerides (mmol/l)	3.27 ± 0.53	3.19 ± 1.01	2.014 ± 0.85	0.0001 S*
Smokers, *n* (%)	30 (69.77)	31 (62)	23 (46)	0.057 NS**

BMI: body mass index; LDL-C: low-density lipoprotein cholesterol; HDL-C: high-density lipoprotein cholesterol. *One-way ANOVA test; **Chi-square test.

**Fig. 3.**
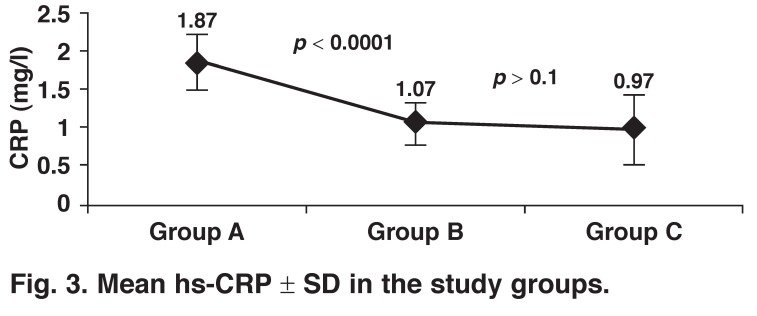
Mean hs-CRP ± SD in the study groups.

Values of hs-CRP were significantly higher in group A (1.87 ± 0.36 mg/l) than in group B (1.07 ± 0.28 mg/l) and group C (0.97 ± 0.45 mg/l) (*p* < 0.0001). There was no significant difference between hs-CRP levels in groups B and C (*p* > 0.1) (Fig. 3).

## Discussion

One of the most important results from this study was that CIMT could predict the presence of coronary artery disease, but it could not predict coronary events. These data are consistent with the findings of other authors. Using radionuclide myocardial perfusion imaging in asymptomatic diabetic subjects, Nair *et al.* found higher carotid IMT values to be highly predictive of ischaemia.^24^

Yoko *et al.* concluded from their study that the addition of max-IMT to conventional risk factors substantially improved the risk stratification for CAD.^25^ Baldassarre *et al.* found that a risk-stratification strategy based on CIMT as an adjunct to the Framingham risk score was a rational approach to prevention of cardiovascular disease.^26^ However, the results of our study did not support the above findings that CIMT is a good predictor of coronary events.

Another important conclusion from our study was that elevated levels of hs-CRP seemed to coincide more with plaque destabilisation in cases of acute coronary syndrome, since hs-CRP levels did not differ significantly in cases with stable CAD from those of healthy individuals. These data are also not consistent with data from other authors. Saudi patients with stable chronic CAD had higher hs-CRP levels compared to healthy individuals.^27^ Khan *et al.* found that patients with chronic stable angina had elevated levels of hs-CRP.^28^

Our results suggest that elevated levels of hs-CRP are manifestations of atherosclerotic plaque instability and a sign of increased risk of acute coronary events. In the study by Ghazala and Mansoor, they confirmed that inflammation can be implicated in the transformation of stable coronary plaque to unstable plaques, rupture and thrombus.^29^ Kadi *et al.* found in patients with stable coronary artery disease and insufficient coronary circulation, serum hs-CRP levels were higher than in patients with adequate coronary collateral circulation.^30^ Zhumin *et al.* found that the values of hs-CRP were higher in patients with unstable carotid atherosclerotic plaques than in patients with stable plaques or without plaques.^31^

According to Ridker *et al.*, hs-CRP has been established as an independent risk factor for future cardiovascular events. It adds prognostic information to the Framingham risk score and at all levels of the metabolic syndrome.^32^ The findings of our study support this opinion.

We believe that the presumed pathophysiological mechanism of atherosclerotic plaque destabilisation in patients with stable coronary artery disease could be via activation of the macrophages in plaques, which leads to secretion of metalloproteinases, cathepsins and collagenases. These enzymes digest the fibrous cap, particularly at the edges, causing the cap to thin and ultimately rupture and initiate plaque thrombosis.

Pre-interventional intravascular ultrasound studies of patients with acute myocardial infarction have shown significantly more plaque rupture in patients with elevated hs-CRP levels, suggesting that this may reflect the inflammatory activity of a ruptured plaque and/or the plausible intensification of focal inflammatory processes that destabilise vulnerable plaques.^33^ Results from this study have shown that measurement of CIMT should be used as a tool to identify individuals with coronary atherosclerosis, but when these changes are associated with increased levels of hs-CRP (in the absence of other causes for the increase in hs-CRP levels) then destabilisation of stable CAD should be considered.

The small sample size was a major limitation of this study. Larger epidemiological studies are needed to clarify the diagnostic value of CIMT to identify CAD, and the usefulness of hS-CRP for the prediction of acute coronary syndromes in cases with increased CIMT.

## Conclusion

Measurement of CIMT is a non-invasive predictor of CAD but it has little prognostic value in predicting CAD events. While hs-CRP is a good predictor of acute coronary events, normal values of hs-CRP do not exclude the presence of stable CAD. The determination of hs-CRP and CIMT together could help in the diagnosis of CAD, and for predicting coronary events. We support the consensual statements for the assessment of CIMT and hs-CRP in individuals who are traditionally considered to be at moderate cardiovascular risk.
